# Differences in diet quality and socioeconomic patterning of diet quality across ethnic groups: cross-sectional data from the HELIUS Dietary Patterns study

**DOI:** 10.1038/s41430-019-0463-4

**Published:** 2019-07-10

**Authors:** Amy Yau, Jean Adams, Martin White, Mary Nicolaou

**Affiliations:** 1 Centre for Diet and Activity Research, Medical Research Council (MRC) Epidemiology Unit, Institute of Metabolic Science, Cambridge Biomedical Campus, University of Cambridge School of Clinical Medicine, Box 285, Cambridge, CB2 0QQ UK; 2 Amsterdam UMC, University of Amsterdam Department of Public Health, Amsterdam Public Health Institute, Meibergdreef 9, 1105 AZ Amsterdam, The Netherlands

**Keywords:** Epidemiology, Epidemiology, Risk factors

## Abstract

**Background/objectives:**

Socioeconomic inequalities in diet quality are consistently reported, but few studies have investigated whether and how such inequalities vary across ethnic groups. This study aimed to examine differences in diet quality and socioeconomic patterning of diet quality across ethnic groups.

**Subjects/methods:**

Cross-sectional data from the HELIUS study were used. Dutch, South-Asian Surinamese, African Surinamese, Ghanaian, Turkish and Moroccan adults (aged 18–70 years) were randomly sampled, stratified by ethnicity. Dietary intake was estimated among a subsample (*n* = 4602) from 200-item, ethnic-specific food frequency questionnaires, and diet quality was assessed using the Dutch Healthy Diet Index 2015 (DHD15-Index). Wald tests were used to compare non-Dutch and Dutch participants. Adjusted linear regression models were used to examine differences in DHD15-Index by three indicators of socioeconomic position: educational level, occupational status and perceived financial difficulties. All analyses were stratified by sex.

**Results:**

Dutch participants had lower median DHD15-Index than most ethnic minority participants (*P* < 0.001). Lower educational level was associated with lower DHD15-Index among Dutch men (*P*_trend_ < 0.0001), South-Asian Surinamese men (*P*_trend_ = 0.01), Dutch women (*P*_trend_ = 0.0001), African Surinamese women (*P*_trend_ = 0.002) and Moroccan women (*P*_trend_ = 0.04). Lower occupational status was associated with lower DHD15-Index in Dutch men, *β* −7.8 (95% CI −11.7, −3.9) and all women (*β* −4.4 to −8.8), except Turkish women. DHD15-Index was not associated with perceived financial difficulties in most groups.

**Conclusions:**

We observed variations in diet quality across ethnic groups. Low socioeconomic position was not consistently associated with poor diet quality in all ethnic groups. This may be due to ethnicity-specific retention of traditional diets, irrespective of socioeconomic position.

## Introduction

Poor diet is a major risk factor for poor health, and dietary risk is not evenly distributed within populations [1]. Socioeconomic gradients in diet quality have been well documented in high-income countries, but much of the data used have poor representation from ethnic minority groups [2, 3]. Prevalence of disease is often higher in ethnic minority groups, and socioeconomic position is on average lower [4], so poorer diet quality among these groups may be expected. Dietary patterns and dietary behaviours differ between ethnic groups [5, 6], which could contribute to ethnic differences in diet quality, and could also modify the relationship between socioeconomic position and diet [7]. These relationships warrant further study, as interventions and policies aiming to improve population diet quality and reduce dietary inequalities should take subgroup differences into consideration.

This study aimed to explore ethnic and socioeconomic inequalities in diet quality across five ethnic groups. First, we examined ethnic differences in the Dutch Healthy Diet Index score 2015 (DHD15-Index), which reflects adherence to the latest Dutch dietary recommendations [8]. We then explored differences in the socioeconomic patterning of diet quality across ethnic groups by examining associations between DHD15-Index and three markers of socioeconomic position: educational level, occupational status and perceived financial difficulties.

## Methods

### Data source and study participants

Participants were from the Healthy Life in an Urban Setting (HELIUS) study, a large cohort of adults (aged 18–70 years) residing in Amsterdam [4]. Participants were randomly sampled, stratified by ethnicity (Dutch, Surinamese, Turkish, Moroccan and Ghanaian) [4, 9]. Full details of the study, including response rates, are available elsewhere [4, 9, 10]. Our study used baseline data, collected between 2011 and 2015, on the subset of participants who completed an ethnic-specific food frequency questionnaire (FFQ) as part of the HELIUS Dietary Patterns study [10, 11]. The semi-quantitative FFQs were developed for the HELIUS study, with ∼200 food items selected based on their percentage contribution to and variance in nutrient intake [11]. This analysis did not include Ghanaian participants, as dietary intake in this group was measured using an FFQ with a different structure [12]. Therefore, we included Dutch, Surinamese, Turkish and Moroccan participants with complete FFQ data. Participants with incomplete socioeconomic position data were excluded (*n* = 95). We further excluded 318 participants due to implausible energy intake using the Willett methods (<800 kcal/day and >4000 kcal/day for men, <500 kcal/day and >3500 kcal/day for women) [13].

The HELIUS study was approved by the Academic Medical Center Ethics Review Board. Written informed consent was obtained from all participants.

### Ethnicity

The municipality register of Amsterdam contains data on country of birth of citizens and of their parents, thus allowing for sampling based on the country of birth indicator of ethnicity [4]. Participants were considered to be of non-Dutch ethnicity if they were born outside of the Netherlands, with at least one parent born outside of the Netherlands (first generation), or born in the Netherlands with both parents born outside the Netherlands (second generation). After data collection, Surinamese participants were further classified according to self-reported ethnic origin (obtained by questionnaire) into ‘African’ or ‘South-Asian’. For the Dutch sample, the study invited people who were born in the Netherlands and whose parents were born in the Netherlands. Participants of this study were classified as Dutch, South-Asian Surinamese, African Surinamese, Turkish or Moroccan. Throughout this article, we refer to ethnicity irrespective of nationality.

### Measuring socioeconomic position

#### Educational level

Participants were split into four categories based on self-reported highest educational attainment: (1) higher (higher vocational and university), (2) intermediate (intermediate vocational and higher secondary schooling), (3) lower (lower vocational and lower secondary schooling) and (4) elementary (never been to school and elementary schooling).

#### Occupational status

Occupational level was classified using the Dutch Standard Occupational Classification 2010 from self-reported occupation. In our analysis, we combined occupational level and employment status to give four categories of occupational status. Three ordinal categories were based on occupational level: (1) higher (scientific and higher occupations), (2) intermediate and (3) lower (elementary and lower occupations). Individuals receiving long-term welfare or seeking employment were also included in the ‘lower’ category. Those with an employment status of ‘unknown/not in workforce’ and no occupational-level data were placed in a fourth heterogeneous category.

#### Perceived financial difficulties

Participants were asked: “During the past year, did you have problems managing your household income?” Four response options were given: “No, no problem at all”, “No problems, but I have to watch what I spend”, “Yes, some problems” and “Yes, lots of problems”. In our analysis, we combined the “Yes” categories.

### Measuring adherence to dietary recommendations and DHD15-Index

Using estimated daily intakes derived from FFQ data and following the methodology described by Looman et al. [8], we calculated DHD15-Index for each participant based on adherence to 13 of the 15 Dutch dietary guidelines: vegetables, fruit, whole grains, legumes, nuts and seeds, dairy, fish, tea, cooking fats and oils, red meat, processed meat, sugar-sweetened beverages (SSBs) and fruit juices, and alcohol (see Supplementary Table S1). Each dietary component was scored between 0 and 10, and the DHD15-Index was a sum of all 13 components, giving a DHD15-Index between 0 and 130. A higher score indicated better diet quality. We were unable to assess compliance with the coffee and salt guidelines due to lack of data.

### Covariates

Covariates associated with diet quality and/or reporting of dietary intake, and that varied across ethnic groups were included in our regression models. The fully adjusted models included potential confounders: age (continuous), marital status (married/cohabiting or not), number of people in the household (continuous), smoking status (current smoker or not), physical activity level (international standard for physical activity^1^ met or not), daily energy intake (continuous) and body mass index (continuous). All covariates were based on self-reported data from the HELIUS questionnaire, except for body mass index which was measured during a physical examination.

### Statistical methods

To examine ethnic differences in diet quality, we calculated age-adjusted medians (lower quartiles and upper quartiles) for DHD15-Index and the individual dietary components for each ethnic group. Medians were used due to the skewness of the data and we adjusted for age due to differences in age distribution between the ethnic groups. Wald tests were used to compare DHD15-Index distribution for the non-Dutch groups with the Dutch group. We used adjusted linear regression models to examine the association between socioeconomic position and DHD15-Index across ethnic groups. We built separate models to explore the associations according to three measures of socioeconomic position: educational level, occupational status and perceived financial difficulties. We obtained *P* for trends by testing equality of means across the socioeconomic strata. A stepwise approach was used to explore the effect of different individual-level, household-level and health-related variables (see Supplementary Tables S2–S7). We stratified all analyses by sex as diet quality and some dietary recommendations differ for men and women (see Supplementary Table S1) [14]. Significance levels were set at a two-tailed *P*-value ≤ 0.05 for all tests. All analyses were conducted in Stata SE 15.

### Sensitivity analyses

In order to understand the effect of one socioeconomic measure on another for diet quality, we ran regression models without mutual adjustment (presented in the main report) and then added other socioeconomic measures to our model individually (presented in Supplementary Tables S8–S13). Educational level and occupational status were moderately correlated (*r* = 0.7), and perceived financial difficulties were weakly associated with educational level (*r* = −0.3) and occupational status (*r* = 0.3).

## Results

### Population characteristics

Overall, 4602 participants were included in this study (see Table 1). Dutch participants tended to have higher socioeconomic position compared with other ethnic groups, with higher educational attainment, higher occupational level and a lower proportion of participants reporting financial difficulties.

**Table 1:** Descriptive characteristics of study population

Characteristics	Men					Women				
	Dutch (*n* = 633)	South-Asian Surinamese (*n* = 395)	African Surinamese (*n* = 298)	Turkish (*n* = 273)	Moroccan (*n* = 258)	Dutch (*n* = 789)	South-Asian Surinamese (*n* = 576)	African Surinamese (*n* = 646)	Turkish (*n* = 305)	Moroccan (*n* = 429)
Age (years), median (lower quartile, upper quartile)	52 (40, 60)	49 (41, 58)	53 (46, 59)	45 (35, 51)	43.5 (35, 53)	49 (35, 59)	49 (41, 56)	51 (43, 57)	42 (32, 49)	39 (30, 49)
Educational level^a^, *n* (%)										
Higher	390 (61.6)	117 (29.6)	69 (23.2)	60 (22.0)	58 (22.5)	492 (62.4)	136 (23.6)	205 (31.7)	65 (21.3)	81 (18.9)
Intermediate	141 (22.3)	111 (28.1)	91 (30.5)	77 (28.2)	84 (32.6)	162 (20.5)	164 (28.5)	234 (36.2)	94 (30.8)	145 (33.8)
Lower	88 (13.9)	120 (30.4)	124 (41.6)	84 (30.8)	59 (22.9)	119 (15.1)	199 (34.6)	186 (28.8)	56 (18.4)	76 (17.7)
Elementary	14 (2.2)	47 (11.9)	14 (4.7)	52 (19.1)	58 (22.5)	16 (2.0)	77 (13.4)	21 (3.3)	90 (29.5)	127 (29.6)
Occupational status^b^, *n* (%)										
Higher	370 (58.5)	100 (25.3)	65 (21.8)	44 (16.2)	44 (17.1)	439 (55.6)	116 (20.1)	161 (24.9)	50 (16.4)	71 (16.6)
Intermediate	150 (23.7)	104 (26.3)	73 (24.5)	49 (18.0)	64 (24.8)	190 (24.1)	177 (30.7)	251 (38.9)	65 (21.3)	93 (21.7)
Lower	88 (13.9)	166 (42.0)	140 (47.0)	164 (60.1)	138 (53.5)	119 (15.1)	238 (41.3)	198 (30.7)	126 (41.3)	148 (34.5)
Unknown/not in workforce	25 (4.0)	25 (6.3)	20 (6.7)	16 (5.9)	12 (4.7)	41 (5.2)	45 (7.8)	36 (5.6)	64 (21.0)	117 (27.3)
Presence of financial difficulties, *n* (%)										
No	327 (51.7)	152 (38.5)	74 (24.8)	44 (16.1)	54 (20.9)	332 (42.1)	132 (22.9)	137 (21.2)	51 (16.7)	87 (20.3)
No, but watch spending	221 (34.9)	140 (35.4)	111 (37.3)	73 (26.7)	85 (33.0)	326 (41.3)	215 (37.3)	240 (37.2)	88 (28.9)	159 (37.1)
Yes	85 (13.4)	103 (26.1)	113 (37.9)	156 (57.1)	119 (46.1)	131 (16.6)	229 (39.8)	269 (41.6)	166 (54.4)	183 (42.7)
Marital status, *n* (% married/cohabiting)	429 (67.8)	228 (57.7)	147 (49.3)	203 (74.4)	196 (76.0)	427 (54.1)	239 (41.5)	168 (26.0)	188 (61.6)	258 (60.1)
Number of people in the household, median (lower quartile, upper quartile)	2 (1, 3)	2 (1, 4)	2 (1, 3)	3 (2, 4)	4 (2, 5)	2 (1, 3)	2 (1, 4)	2 (1, 3)	3 (2, 4)	4 (2, 5)
Current smoking status, *n* (% yes)	149 (23.5)	135 (34.2)	101 (33.9)	81 (29.7)	48 (18.6)	174 (22.1)	95 (16.5)	117 (18.1)	82 (26.9)	20 (4.7)
Physical activity norm met^c^, *n* (% yes)	459 (72.5)	216 (54.7)	196 (65.8)	141 (51.7)	150 (58.1)	601 (76.2)	291 (50.5)	362 (56.0)	121 (39.7)	167 (38.9)
Energy intake (kcal), median (lower quartile, upper quartile)	2375 (2004, 2887)	2130 (1752, 2606)	2372 (1833, 2911)	2329 (1837, 2934)	2394 (1755, 3018)	1960 (1629, 2298)	1743 (1362, 2150)	1816 (1391, 2313)	1871 (1474, 2441)	1814 (1399, 2279)
Body mass index (kg/m^2^), median (lower quartile, upper quartile)	24.8 (22.7, 27.4)	25.3 (23.1, 27.6)	26.3 (23.8, 28.8)	27.9 (25.2, 30.4)	26.8 (24.6, 29.4)	23.5 (21.5, 26.2)	26.5 (23.2, 29.8)	28.2 (24.9, 32.2)	27.2 (24.1, 32.0)	27.5 (23.8, 31.8)
Presence of chronic disease^d^, *n* (% yes)	96 (15.2)	140 (35.4)	54 (18.1)	72 (26.4)	86 (33.3)	92 (11.7)	192 (33.3)	181 (28.0)	95 (31.2)	111 (25.9)

^a^Higher = higher vocational schooling or university, Intermediate = intermediate vocational schooling or intermediate/higher secondary schooling, Lower = lower vocational schooling or lower secondary schooling, Elementary = never been to school or elementary schooling

^b^Higher = higher-grade professional occupations, Intermediate = lower-grade professional and routine non-manual occupations, Lower = skilled and unskilled manual occupations, and unemployed (seeking work or receiving social benefits), Unknown/not in workforce = unknown occupational level (employed but no occupation level available) and not in workforce (retired, student, homemaker or incapacitated to work)

^c^Met the Short Questionnaire to Assess Health-Enhancing Physical Activity (SQASH) international norm for physical activity (≥30 min of moderate- and high-intensity activity per day on at least 5 days per week)

^d^Presence of one or more chronic disease (diabetes, cardiovascular disease, myocardial infarction or cancer)

### DHD15-Index

**Fig. 1:**
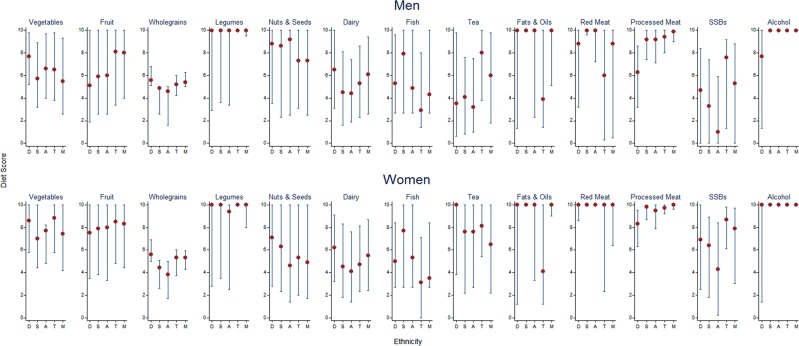
Age-adjusted median (lower quartile, upper quartile) DHD15-Index for individual food group components by ethnicity and sex. SSBs, sugar-sweetened beverages. D Dutch, S South-Asian Surinamese, A African Surinamese, T Turkish, M Moroccan

The distribution of DHD15-Index varied by ethnicity, with Dutch and African Surinamese participants having the lowest age-adjusted median (*P* < 0.0001) (see Table 2). Figure 1 shows age-adjusted median (lower quartile, upper quartile) scores for individual dietary components. There were differences between the ethnic groups for all dietary components, except for nuts and seeds in men (see Supplementary Table S14) and fruit, legumes, red meat and alcohol in women (see Supplementary Table S15). Dutch men had higher vegetable intake than men from other ethnic groups, but the lowest fruit intake. Adherence to the whole-grain and dairy recommendations was moderately low in all ethnic groups, but the highest among Dutch participants. Fish intake was low to moderate overall, with South-Asian Surinamese scoring the highest. All ethnic groups had a healthy ratio of liquid/soft fats to solid fats used in cooking, except for Turkish participants. Turkish men scored particularly poorly for red meat, whilst Dutch participants scored the worst for processed meat. Scores for SSBs and fruit juice were especially poor among African Surinamese participants. All groups scored highly for alcohol, but variation in scores was high in Dutch participants and scores were the lowest among Dutch men.

**Table 2:** Age-adjusted median (lower quartile, upper quartile) DHD15-Index score by ethnicity and sex

	Dutch	South-Asian Surinamese	African Surinamese	Turkish	Moroccan	Pearson’s F statistic (*P*-value)
**Overall**	83.3 (71.5, 94.8)	87.0 (75.8, 98.0)	82.5 (71.7, 92.6)	88.5 (79.1, 97.5)	89.4 (79.2, 100.4)	18.10 (<0.0001)
**Men**	78.6 (67.8, 90.2)	83.3 (72.3, 93.9)	77.4 (67.2, 88.6)	85.4 (76.8, 95.0)	87.5 (76.1, 97.9)	13.78 (<0.0001)
**Women**	86.9 (76.0, 97.7)	90.4 (78.7, 100.4)	84.4 (73.4, 94.4)	90.8 (81.7, 98.5)	90.4 (80.8, 101.1)	10.19 (<0.0001)

### Socioeconomic inequalities in DHD15-Index

#### Educational level

**Fig. 2:**
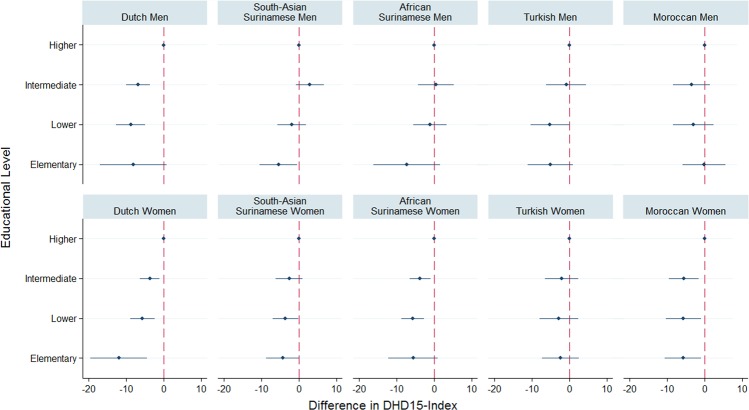
Differences in DHD15-Index by educational level, stratified by ethnicity and sex. Reference group: higher educational level. Regression models adjusted for age, marital status, number of people in the household, smoking status, meeting of physical activity recommendation, energy intake, presence of one or more chronic disease and body mass index

Figure 2 shows the β-coefficients (95% CIs) for the fully adjusted linear regression models (model 4) examining associations between educational level and DHD15-Index, stratified by ethnicity and sex (see Supplementary Tables S2 and S3 for further details, including the stepwise models).

An educational gradient in DHD15-Index was observed among Dutch men, with those less educated having a lower DHD15-Index (*P*_trend_ < 0.0001). South-Asian Surinamese men with elementary education had lower DHD15-Index than those with higher education (*P*_trend_ = 0.01). No educational differences were observed in men from other ethnic groups. Lower educational level was associated with lower DHD15-Index among Dutch women (*P*_trend_ = 0.0001). African Surinamese women with lower and intermediate educational level had lower DHD15-Index compared with those with higher educational level (*P*_trend_ = 0.002). Moroccan women in all educational groups had lower DHD15-Index compared with the higher educational-level group (*P*_trend_ = 0.04). No educational differences in DHD15-Index were observed for South-Asian Surinamese or Turkish women.

#### Occupational status

**Fig. 3:**
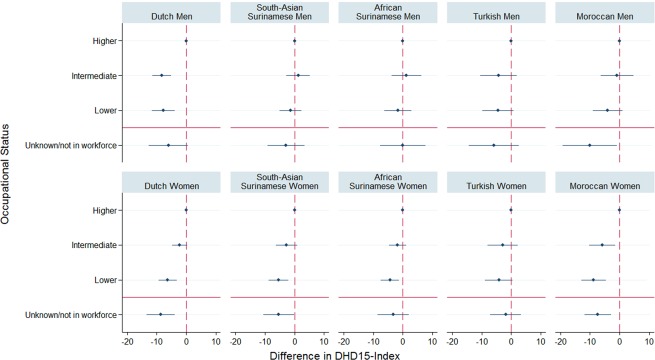
Differences in DHD15-Index by occupational status, stratified by ethnicity and sex. Reference group: higher occupational level. Ordinal occupational levels above the red line, unknown/not in the workforce group below the red line. Regression models adjusted for age, marital status, number of people in the household, smoking status, meeting of physical activity recommendation, energy intake, presence of one or more chronic disease and body mass index

Figure 3 shows the results of the fully adjusted linear regression models examining associations between occupational status and DHD15-Index (further information in Supplementary Tables S4 and S5). Dutch men with intermediate and elementary occupations had lower DHD15-Index than those with higher occupational status (*P*_trend_ < 0.0001). No occupational differences were seen among men from other ethnic groups, but those in the unknown/not in the workforce group had lower DHD15-Index compared to those with higher occupation status among Moroccan men. Women with elementary-level occupations had lower DHD15-Index than those with higher-level occupations among Dutch (*P*_trend_ < 0.0001), South-Asian Surinamese (*P*_trend_ = 0.01), African Surinamese (*P*_trend_ = 0.04) and Moroccan (*P*_trend_ = 0.001) participants. No association was observed in Turkish women.

#### Perceived financial difficulties

**Fig. 4:**
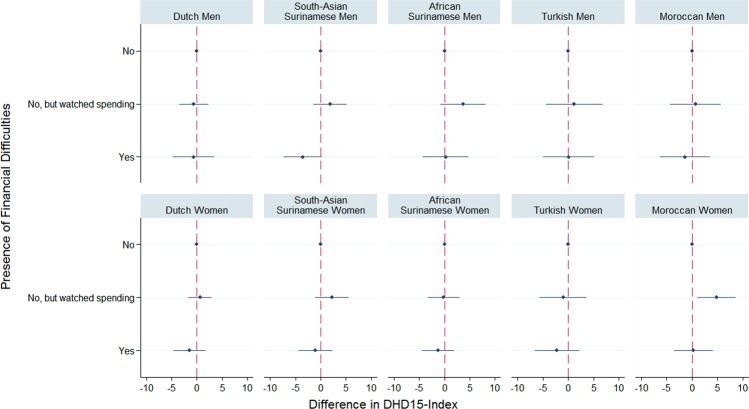
Differences in DHD15-Index by perceived financial difficulties, stratified by ethnicity and sex. Reference group: no financial difficulties. Regression models adjusted for age, marital status, number of people in the household, smoking status, meeting of physical activity recommendation, energy intake, presence of one or more chronic disease and body mass index

Figure 4 presents the results of the fully adjusted linear regression models examining associations between perceived financial difficulties and DHD15-Index (more details in Supplementary Tables S6 and S7). No differences in DHD15-Index were observed in men by perceived financial difficulties in any of the ethnic groups. For women, Moroccan participants who reported that they did not have financial difficulties but did watch their spending had a higher DHD15-Index than those who reported no financial difficulties at all (*P*_trend_ = 0.01).

### Sensitivity analyses

In our sensitivity analyses, we mutually adjusted our regression models for socioeconomic measures (see Supplementary Tables S8–S13). In general, similar trends were observed; however, most associations were reduced. Educational differences in DHD15-Index remained for Dutch men and African Surinamese women only. This suggests that the association between educational level and diet quality in the other groups may have been largely through occupational status. Occupational differences in DHD15-Index reduced for most groups once educational level was adjusted for, which could be partly mediating this relationship. However, occupational differences in DHD15-Index remained significant for Dutch men, South-Asian Surinamese women and Moroccan women.

## Discussion

We found ethnic differences in diet quality, operationalised as the DHD15-Index, with most ethnic groups having higher diet quality than the Dutch. Ethnic differences were observed for the intake of most food groups; thus, variation in diet quality was not driven by any specific food group. Educational differences in DHD15-Index were clearest among Dutch participants, and also observed in South-Asian Surinamese men, African Surinamese women and Moroccan women. Occupational differences in diet quality were seen among Dutch men and in most ethnic groups for women. These differences, as expected, favoured those of higher socioeconomic position. Differences in DHD15-Index by perceived financial difficulties were not seen in most groups.

### Strengths and limitations

The HELIUS study provided large samples of five ethnic groups, with dietary data through ethnic-specific FFQs and details of socioeconomic position through three proxy measures: educational level, occupational status and perceived financial difficulties. This offered a rare opportunity to explore diet quality across ethnic groups and in relation to a variety of measures of socioeconomic position. FFQs are one of the best ways of capturing habitual dietary intake in ethnically diverse populations [11]. However, as with all self-reported data, FFQs are subject to social desirability bias. FFQs also yield higher DHD15-Index compared with 24-h recalls; therefore, absolute DHD15-Index may be inflated [8]. DHD15-Index is associated with body mass index and all-cause mortality [8, 15], but further research is needed to explore whether there are ethnic differences in these associations.

Our observations may be relevant to other contexts with similar ethnic groups; however, the specificities of the Dutch migration history may limit generalisability of the findings. Nonetheless, ethnic differences in diet quality have been reported elsewhere, although most studies are from the United States and find that ethnic minority groups have poorer diet quality than the ethnic majority group [16, 17]. The educational gradient and occupational differences in diet quality observed in some groups in this study are consistent with many previous studies [18, 19]. To our knowledge, few studies have compared the association between socioeconomic position and diet quality across ethnic groups. Those that have, found socioeconomic and ethnic inequalities in diet independently, and interaction between the two variables [7, 20, 21].

### Interpretation of findings and implications for policy

Lower overall socioeconomic position was seen among ethnic minority groups compared with the Dutch group. However, most ethnic minority groups had higher DHD15-Index than the Dutch group. Socioeconomic gradients in diet quality were also not seen in all ethnic groups. This could suggest resilience to the negative consequence of lower educational level and occupational status for diet quality amongst these groups. Further understanding this relationship could help to improve diet quality in whole populations. Factors associated with diet in ethnic minority groups can be clustered into seven themes: migration context; social and cultural environment; food beliefs and perceptions; accessibility of food; the body; psychosocial; social and material resources [22]. These likely impact on differences in the overall diet quality between ethnic groups, and could also explain differences in socioeconomic patterning of diet quality between ethnic groups.

As populations around the world become more ethnically diverse, it is important to recognise that many dietary patterns can be supportive of good diet quality, and dietary public health should value traditional food cultures and variation in dietary habits. Global trends of urbanisation and economic growth are linked to nutritional and epidemiological transitions, and increased prevalence of non-communicable diseases [23]. For migrants, dietary acculturation, whereby migrant populations adopt dietary habits of their host country over time, may also worsen diet quality and health outcomes [24]. Eighty-two percent of the ethnic minority participants in our study were first-generation immigrants. Retention of elements of traditional diets could explain higher diet quality among migrants compared with Dutch participants, assuming that the Western diet is less healthy [25]. This could also explain inconsistent socioeconomic patterning of diet quality among ethnic minority groups, if components of the traditional diets are retained as a way of expressing cultural identity, regardless of socioeconomic position [5]. Cultural expectation of hospitality [26], and the food preferences of family and friends, especially in collectivist cultures, may also prevent or slow shifts in dietary habits from the traditional diet. Alternatively, the lack of association seen could be due to the proxy measures of socioeconomic position requiring different interpretations depending on ethnicity. The same objective educational level could be associated with different social and environmental contexts and job prospects for different ethnic groups.

Whilst DHD15-Index focuses on diet quality as a whole, we saw that scores for individual components varied substantially across ethnic groups too. This suggests that the dietary components that need most attention differ by ethnicity, and this knowledge could be useful in developing dietary interventions and tailoring dietary advice. Consistent with the notion of the Western diet [25], we found higher processed meat and alcohol intake, and lower fruit intake (significant only in men) among Dutch participants, but more favourable intakes of dairy and whole grains compared with the other ethnic groups. Turkish participants scored substantially worse for cooking oils and fats compared with other ethnic groups, and African Surinamese participants scored particularly poorly for SSBs and fruit juice. On the other hand, guidelines were well met for some dietary components. For example, the median score was 10 out of 10 for legumes, cooking fats and oils and alcohol for most groups.

In our study, perceived financial difficulties were not associated with diet quality for most groups. This was an unexpected finding, as previous studies have shown an association between diet cost and diet quality [27, 28]. There could be various explanations for the lack of association in our analysis. The question used may have been a poor measure of financial difficulties. The only significant difference in diet was between those reporting that they had no financial difficulties but were careful with spending and those with no financial difficulties at all. This could be because participants who were careful with finances were also more likely to be careful with other aspects of their lives, including diet, and the two groups may not have differed in terms of financial resources. Furthermore, short-term financial difficulty could be a poor measure of socioeconomic position, with educational level and occupational status potentially providing more stable and long-term proxies [20]. The presence of educational and occupational, but not financial, differences in diet quality may also suggest that the mechanism driving socioeconomic differences in diet quality is psychosocial rather than material. Alternatively, diet cost may not be a barrier to good diet quality among Amsterdam residents, perhaps due to low food costs, a healthy food environment and/or good support for those who are financially struggling to meet their dietary needs [29].

## Conclusions

Diet quality varied across ethnic groups, with better diet quality in most ethnic minority groups compared with the majority ethnic group. Nonetheless, diet quality was suboptimal in all groups and improvement of diet should remain a public health priority for the whole population. Low socioeconomic position was only associated with poorer diet quality in some ethnic groups, indicating that socioeconomic deprivation is not a universal indicator of poor diet quality. Similarities in diet quality across the socioeconomic spectrum in some groups may be due to retention of elements of traditional diets, irrespective of socioeconomic position. Future dietary interventions should consider the role of culture and tradition in maintaining dietary habits.

## Supplementary information

Supplementary Material.

## Footnotes

1 Short Questionnaire to Assess Health Enhancing Physical Activity (SQUASH) standard: ≥30 min of moderate- or high-intensity activity per day on at least 5 days per week.
