# Supplementary material for: Differences in diet quality and socioeconomic patterning of diet quality across ethnic groups: cross-sectional data from the HELIUS Dietary Patterns study
Source: Eur J Clin Nutr. 2019 Jul 10;74(3):387–96. doi: 10.1038/s41430-019-0463-4 (PMC7062636; doi:10.1038/s41430-019-0463-4)
Supplement: Supplementary file 1 — Supplementary Material. [file 41430_2019_463_MOESM1_ESM.docx]

**Supplementary Table S1:** DHD15-Index dietary components included in this study

| **Dietary Component** | **Inclusion/Exclusion/Restriction** | **Recommendation** | **Threshold (0 point)** | **Cut-off (10 points)** |
| --- | --- | --- | --- | --- |
| Vegetables | **Included:** frozen and canned, peas, salad  **Excluded:** legumes, potatoes | Increase consumption | 0g/day | ≥200g/day |
| Fruit | **Included:** fresh fruit  **Excluded:** dried fruit, apple sauce, fruit juice | Increase consumption | 0g/day | ≥200g/day |
| Wholegrains* | **Included:** staple cereal products e.g. bread, couscous, muesli, pasta, rice  **Excluded:** snack cereal products e.g. biscuits | Increase consumption | a) 0g/day | 90g/day (5 points) |
|  |  | Replace refined grains with wholegrains | b) wholegrain to refined grain ratio ≤0.7 | wholegrain to refined grain ratio ≥11 (5 points) |
| Legumes | **Included:** pulses, lentils, beans, chickpeas  **Excluded:** peas, peanuts | Increase consumption | 0g/day | ≥10g/day |
| Nuts & seeds | Nuts and seeds only | Increase consumption | 0g/day | ≥15g/day |
| Dairy | **Included:** milk, milk products, yoghurt, cheese, cream, custard, porridge prepared with dairy  **Restricted:** Up to 40g/day of cheese was included | Maintain consumption within optimal range | 0g/day (lower threshold) | 300g/day-450g/day |
|  |  |  | ≥750g/day (upper threshold) |  |
| Fish | **Included:** all oily fish  **Restricted:** Up to 4g/day of lean fish and crustaceans/molluscs was included | Increase consumption | 0g/day | ≥15g/day |
| Tea | Black or green tea only | Increase consumption | 0g/day | ≥450g/day |
| Cooking fats & oils | **Solid:** butter, hard margarine  **Liquid/soft:** oils, soft margarine, halvarine | Replace solid fats with liquid/soft fats | Liquid/soft to solid ratio ≤0.6 | Liquid/soft to solid ratio ≥13 |
| Red meat | **Included:** beef, pork, duck, pheasant, offal, game | Limit consumption | ≥100g/day | ≤45g/day |
| Processed meat | **Included:** red and white processed meat | Limit consumption | ≥50g/day | 0g/day |
| SSBs & fruit juices | **Included:** sugar-sweetened soft drinks, sugar-sweetened dairy drinks, fruit juice | Limit consumption | ≥250g/day | 0g/day |
| Alcohol |  | Limit consumption | Men: ≥30g ethanol/day  Women: ≥20g/d ethanol/day | ≤10g ethanol/day |
| DHD15-Index | Sum of all 13 food groups | N/A | Minimum score: 0 | Maximum score: 130 |
| SSBs=sugar-sweetened beverages  *products containing ≥25% wholegrain flour | | | | |

Participants were scored between 0 and 10 for each component based on their estimated intake. The cut-off value was awarded 10 out of 10 and the threshold value was given 0 out of 10, with intakes in between scored proportionately. Most food groups use a maximum or minimum as the cut-off value and threshold value. However for dairy, those who consumed 300g/day-450g/day achieved 10 out of 10, and those who ate less or more received a lower score. Some food items were limited in how much they could contribute to meeting a recommendation (lean fish, crustaceans/molluscs and cheese).

**Supplementary Table S2:** Beta-coefficients (95% confidence intervals) from adjusted linear regression models for association between DHD15-Index and educational level in men

|  | | **Dutch**  β (95% CI) | **South-Asian Surinamese**  β (95% CI) | **African Surinamese**  β (95% CI) | **Turkish**  β (95% CI) | **Moroccan**  β (95% CI) |
| --- | --- | --- | --- | --- | --- | --- |
| **Model 1** | Higher | REF | REF | REF | REF | REF |
|  | Intermediate | -8.4 (-11.6, -5.2)*** | 3.3 (-0.5, 7.1) | 0.9 (-3.9, 5.7) | -3.3 (-8.5, 1.8) | -1.6 (-6.7, 3.5) |
|  | Lower | -10.3 (-14.3, -6.3)*** | -2.5 (-6.3, 1.3) | -0.4 (-4.9, 4.1) | -6.6 (-11.7, -1.5)* | -2.7 (-8.3, 2.8) |
|  | Elementary | -11.0 (-20.0, -2.0)* | -6.2 (-11.2, -1.1)* | -7.9 (-16.8, 0.9) | -7.3 (-13.2, -1.3)* | 0.7 (-5.2, 6.5) |
| **Model 2** | Higher | REF | REF | REF | REF | REF |
|  | Intermediate | -8.3 (-11.5, -5.0)*** | 3.3 (-0.5, 7.2) | 0.8 (-4.0, 5.6) | -1.8 (-7.2, 3.5) | -1.8 (-6.9, 3.3) |
|  | Lower | -10.3 (-14.3, -6.3)*** | -2.5 (-6.3, 1.4) | -0.9 (-5.5, 3.6) | -5.9 (-11.1, -0.6)* | -2.9 (-8.5, 2.7) |
|  | Elementary | -11.2 (-20.2, -2.2)* | -6.1 (-11.1, -1.0)* | -9.2 (-18.1, -0.3)* | -6.3 (-12.3, -0.3)* | 0.4 (-5.5, 6.4) |
| **Model 3** | Higher | REF | REF | REF | REF | REF |
|  | Intermediate | -7.6 (-10.8, -4.5)*** | 2.9 (-0.8, 6.6) | 0.9 (-3.9, 5.6) | -1.1 (-6.3, 4.0) | 2.8 (-7.9, 2.2) |
|  | Lower | -9.5 (-13.4, -5.6)*** | -1.7 (-5.6, 2.1) | -0.4 (-4.9, 4.1) | -5.3 (-10.4, -0.2)* | -2.8 (-8.3, 2.7) |
|  | Elementary | -10.0 (-18.8, -1.3)* | -5.3 (-10.3, -0.3)* | -7.3 (-16.2, 1.5) | -5.3 (-11.2, 0.6) | 0.4 (-5.4, 6.2) |
| **Model 4** | Higher | REF | REF | REF | REF | REF |
|  | Intermediate | -6.8 (-10.0, -3.6)*** | 3.0 (-0.8, 6.7) | 0.5 (-4.3, 5.2) | -0.8 (-6.1, 4.5) | -3.5 (-8.4, 1.5) |
|  | Lower | -8.7 (-12.7, -4.8)*** | -1.9 (-5.7, 2.0) | -1.1 (-5.6, 3.4) | -5.1 -10.3, 0.1) | -2.9 (-8.3, 2.5) |
|  | Elementary | -8.0 (-16.9, 0.8) | -5.4 (-10.4, -0.4)* | -7.2 (-16.1, 1.6) | -5.0 (-11.1, 1.1) | -0.0 (-5.7, 5.6) |
|  | P_trend_ | <0.0001*** | 0.01** | 0.35 | 0.11 | 0.36 |
| β =beta coefficient, CI=confidence interval.  Model 1 adjusted for age. Model 2: model 1 + adjusted for marital status, number of people in the household. Model 3: model 2 + adjusted for smoking status, physical activity, energy intake. Model 4: adjusted for model 3 + presence of one or more chronic disease, body mass index.  * *P*≤0.05, ***P*≤0.01, ****P*≤0.001 | | | | | | |

**Supplementary Table S3:** Beta-coefficients (95% confidence intervals) from adjusted linear regression models for association between DHD15-Index and educational level in women

|  | | **Dutch**  β (95% CI) | **South-Asian Surinamese**  β (95% CI) | **African Surinamese**  β (95% CI) | **Turkish**  β (95% CI) | **Moroccan**  β (95% CI) |
| --- | --- | --- | --- | --- | --- | --- |
| **Model 1** | Higher | REF | REF | REF | REF | REF |
|  | Intermediate | -4.8 (-7.5, -2.1)*** | -1.7 (-5.2, 1.8) | -4.1 (-6.8, -1.3)** | -2.6 (-7.0, 1.8) | -4.7 (-8.6, -0.7)* |
|  | Lower | -8.3 (-11.5, -5.0)*** | -2.3 (-5.8, 1.2) | -5.9 (-8.8, -2.9)*** | -4.4 (-9.4, 0.5) | -4.8 (-9.5, -0.1)* |
|  | Elementary | -15.8 (-23.4, -8.1)*** | -3.9 (-8.4, 0.6) | -5.4 (-12.0, 1.1) | -2.5 (-7.2, 2.2) | -4.4 (-9.1, 0.3) |
| **Model 2** | Higher | REF | REF | REF | REF | REF |
|  | Intermediate | -4.7 (-7.4, -2.0)*** | -1.5 (-5.1, 2.0) | -4.0 (-6.8, -1.3)** | -3.4 (-7.8, 1.1) | -5.1 (-9.1, -1.1)* |
|  | Lower | -8.3 (-11.5, -5.0)*** | -2.5 (-6.0, 1.0) | -6.0 (-8.9, -3.0)*** | -5.4 (-10.4, -0.4)* | -5.8 (-10.6, -1.1)* |
|  | Elementary | -15.8 (-23.4, -8.1)*** | -3.7 (-8.2, 0.8) | -5.6 (-12.2, 1.0) | -3.6 (-8.3, 1.2) | -5.3 (-10.1, -0.5)* |
| **Model 3** | Higher | REF | REF | REF | REF | REF |
|  | Intermediate | -3.9 (-6.6, -1.3)** | -2.3 (-5.8, 1.2) | -3.6 (-6.4, -0.8)* | -2.7 (-7.0, 1.7) | -5.4 (-9.4, -1.4)** |
|  | Lower | -6.3 (-9.5, -3.1)*** | -3.3 (-6.7, 0.1) | -5.4 (-8.4, -2.5)*** | -3.8 (-8.8, 1.1) | -5.5 (-10.1, -0.8)* |
|  | Elementary | -12.7 (-20.2, -5.2)*** | -3.8 (-8.2, 0.6) | -5.2 (-11.8, 1.3) | -3.3 (-8.0, 1.3) | -5.4 (-10.1, -0.6)* |
| **Model 4** | Higher | REF | REF | REF | REF | REF |
|  | Intermediate | -3.7 (-6.3, -1.0)** | -2.5 (-6.1, 1.0) | -3.7 (-6.4, -0.9)** | -2.0 (-6.5, 2.4) | -5.5 (-9.5, -1.6)** |
|  | Lower | -5.7 (-8.9, -2.4)*** | -3.6 (-7.0, -0.1)* | -5.7 (-8.7, -2.7)*** | -2.8 (-7.9, 2.4) | -5.6 (-10.3, -0.9)* |
|  | Elementary | -11.9 (-19.4, -4.4)** | -4.3 (-8.7, 0.1) | -5.6 (-12.1, 1.0) | -2.3 (-7.2, 2.6) | -5.7 (-10.6, -0.9)* |
|  | P_trend_ | 0.0001*** | 0.16 | 0.002** | 0.72 | 0.04* |
| β =beta coefficient, CI=confidence interval.  Model 1 adjusted for age. Model 2: model 1 + adjusted for marital status, number of people in the household. Model 3: model 2 + adjusted for smoking status, physical activity, energy intake. Model 4: adjusted for model 3 + presence of one or more chronic disease, body mass index.  * *P*≤0.05, ***P*≤0.01, ****P*≤0.001 | | | | | | |

**Supplementary Table S4:** Beta-coefficients (95% confidence intervals) from adjusted linear regression models for association between DHD15-Index and occupational status in men

|  | | **Dutch**  β (95% CI) | **South-Asian Surinamese**  β (95% CI) | **African Surinamese**  β (95% CI) | **Turkish**  β (95% CI) | **Moroccan**  β (95% CI) |
| --- | --- | --- | --- | --- | --- | --- |
| **Model 1** | Higher | REF | REF | REF | REF | REF |
|  | Intermediate | -8.8 (-12.0, -5.6)*** | 0.7 (-3.3, 4.8) | 0.8 (-4.4, 6.0) | -6.6 (-12.7, -0.4)* | 1.1 (-4.7, 6.8) |
|  | Elementary | -8.8 (-12.7, -4.8)*** | -1.8 (-5.5, 1.9) | -1.2 (-5.8, 3.3) | -6.6 (-11.7. -1.4)* | -3.0 (-8.2, 2.1) |
|  | Unknown/not in workforce | -6.1 (-13.0, 0.8) | -2.7 (-9.2, 3.9) | 0.7 (-7.0, 8.5) | -8.2 (-16.8, 0.4) | -9.1 (-18.6, 0.5) |
| **Model 2** | Higher | REF | REF | REF | REF | REF |
|  | Intermediate | -8.8 (-12.1, -5.6)*** | 0.9 (-3.2, 5.0) | 0.9 (-4.3, 6.1) | -5.7 (-11.9. 0.6) | 1.0 (-4.7, 6.7) |
|  | Elementary | -8.4 (-12.4, -4.5)*** | -1.7 (-5.5, 2.0) | -1.8 (-6.4, 2.8) | -5.7 (-11.0, -0.4)* | -3.2 (-8.4, 2.0) |
|  | Unknown/not in workforce | -6.1 (-12.9, 0.8) | -2.6 (-9.2, 3.9) | 1.0 (-6.7, 8.8) | -6.8 (-15.5, 1.9) | -8.9 (-18.5, 0.7) |
| **Model 3** | Higher | REF | REF | REF | REF | REF |
|  | Intermediate | -9.0 (-12.1, -5.9)*** | 1.2 (-2.7, 5.2) | 1.6 (-3.5, 6.7) | -4.5 (-10.6, 1.7) | -0.0 (-5.6, 5.6) |
|  | Elementary | -8.7 (-12.6, -4.8)*** | -1.5 (-5.2, 2.2) | -1.1 (-5.7, 3.4) | -4.8 (-10.0, 0.5) | -3.8 (-8.9, 1.3) |
|  | Unknown | -6.7 (-13.4, -0.0)* | -2.7 (-9.0, 3.6) | 0.6 (-7.0, 8.3) | -5.9 (-14.4, 2.6) | -9.4 (-18.8, 0.1) |
| **Model 4** | Higher | REF | REF | REF | REF | REF |
|  | Intermediate | -8.3 (-11.4, -5.1)*** | 1.2 (2.8, 5.2) | 1.2 (-3.9, 6.3) | -4.3 (-10.5, 1.9) | -0.8 (-6.4, 4.8) |
|  | Elementary | -7.8 (-11.7, -3.9)*** | -1.4 (-5.0, 2.3) | -1.7 (-6.2, 2.9) | -4.5 (-9.8, 0.8) | -4.0 (-9.0, 1.0) |
|  | Unknown/not in workforce | -6.1 (-12.7, 0.6) | -2.9 (-9.2, 3.4) | -0.0 (-7.7, 7.6) | -5.9 (-14.4, 2.7) | -10.1 (-19.3, -0.8)* |
|  | P_trend_ | <0.0001******* | 0.40 | 0.60 | 0.36 | 0.09 |
| β =beta coefficient, CI=confidence interval.  Model 1 adjusted for age. Model 2: model 1 + adjusted for marital status, number of people in the household. Model 3: model 2 + adjusted for smoking status, physical activity, energy intake. Model 4: adjusted for model 3 + presence of one or more chronic disease, body mass index.  * *P*≤0.05, ***P*≤0.01, ****P*≤0.001 | | | | | | |

**Supplementary Table S5:** Beta-coefficients (95% confidence intervals) from adjusted linear regression models for association between DHD15-Index and occupational status in women

|  | | **Dutch**  β (95% CI) | **South-Asian Surinamese**  β (95% CI) | **African Surinamese**  β (95% CI) | **Turkish**  β (95% CI) | **Moroccan**  β (95% CI) |
| --- | --- | --- | --- | --- | --- | --- |
| **Model 1** | Higher | REF | REF | REF | REF | REF |
|  | Intermediate | -3.3 (-5.9, -0.6)* | -1.9 (-5.5, 1.7) | -2.0 (-4.9, 0.9) | -4.4 (-9.5, 0.8) | -5.6 (-10.0, -1.1)* |
|  | Elementary | -8.0 (-11.1, -4.9)*** | -4.8 (-8.2, -1.4)** | -4.6 (-7.7, -1.5)** | -5.0 (-9.6, -0.4)* | -8.6 (-12.8, -4.4)*** |
|  | Unknown/not in workforce | -8.4 (-13.3, -3.5)*** | -4.9 (-10.2, 0.3) | -3.4 (-8.8, 2.0) | -2.2 (-7.3, 3.0) | -6.6 (-11.1, -2.2)** |
| **Model 2** | Higher | REF | REF | REF | REF | REF |
|  | Intermediate | -3.3 (-5.9, -0.7)* | -1.9 (-5.5, 1.7) | -2.0 (-4.9, 0.9) | -4.8 (-9.9, 0.3) | -5.8 (-10.2, -1.4)** |
|  | Elementary | -7.9 (-11.1, -4.8)*** | -4.6 (-8.1, -1.2)** | -4.6 (-7.7, -1.5)** | -5.3 (-9.9, -0.7)* | -9.0 (13.2, -4.8)*** |
|  | Unknown/not in workforce | -8.5 (-13.5, -3.6)*** | -4.6 (-9.9, 0.8) | -3.5 (-8.9, 1.9) | -2.4 (-7.6, 2.8) | -7.4 (-11.9, -2.9)*** |
| **Model 3** | Higher | REF | REF | REF | REF | REF |
|  | Intermediate | -2.7 (-5.2, -0.2)* | -2.6 (-6.1, 1.0) | -1.7 (-4.6, 1.2) | -3.8 (-8.8, 1.1) | -5.7 (-10.1, -1.3)* |
|  | Elementary | -6.9 (-9.9, -3.8)*** | -5.0 (-8.4, -1.6)** | -4.0 (-7.1, -1.0)** | -5.1 (-9.6, -0.6)* | -8.6 (-12.8, -4.5)*** |
|  | Unknown/not in workforce | -8.8 (-13.6, -4.1)*** | -4.9 (-10.1, 0.3) | -3.0 (-8.3, 2.3) | -2.8 (-7.8, 2.3) | -7.1 (-11.5, -2.6)** |
| **Model 4** | Higher | REF | REF | REF | REF | REF |
|  | Intermediate | -2.2 (-4.8, 0.3) | -2.7 (-6.2, 0.9) | -1.8 (-4.7, 1.1) | -2.9 (-8.1, 2.2) | -5.8 (-10.2, -1.4)** |
|  | Elementary | -6.3 (-9.3, -3.2)*** | -5.4 (-8.8, -2.0)** | -4.4 (-7.5, -1.3)** | -4.2 (-8.8, 0.5) | -8.8 (-12.9, -4.6)*** |
|  | Unknown/not in workforce | -8.7 (-13.5, -4.0)*** | -5.4 (-10.6, -0.2)* | -3.3 (-8.6, 2.0) | -1.9 (-7.1, 3.3) | -7.4 (-11.9, -2.9)*** |
|  | P_trend_ | <0.0001*** | 0.01** | 0.04* | 0.33 | 0.001*** |
| β =beta coefficient, CI=confidence interval.  Model 1 adjusted for age. Model 2: model 1 + adjusted for marital status, number of people in the household. Model 3: model 2 + adjusted for smoking status, physical activity, energy intake. Model 4: adjusted for model 3 + presence of one or more chronic disease, body mass index.  * *P*≤0.05, ***P*≤0.01, ****P*≤0.001 | | | | | | |

**Supplementary Table S6:** Beta-coefficients (95% confidence intervals) from adjusted linear regression models for association between DHD15-Index and financial difficulty status in men

|  | | **Dutch**  β (95% CI) | **South-Asian Surinamese**  β (95% CI) | **African Surinamese**  β (95% CI) | **Turkish**  β (95% CI) | **Moroccan**  β (95% CI) |
| --- | --- | --- | --- | --- | --- | --- |
| **Model 1** | No | REF | REF | REF | REF | REF |
|  | No, but careful | -1.8 (-4.7, 1.2) | 1.3 (-2.1, 4.7) | 3.4 (-1.1, 7.9) | 2.3 (-3.4, 8.0) | 1.5 (-3.6, 6.7) |
|  | Yes | -3.0 (-7.2, 1.1) | -3.4 (-7.1, 0.2) | 0.4 (-4.1, 4.9) | -0.3 (-5.4, 4.9) | -1.4 (-6.4, 3.6) |
| **Model 2** | No | REF | REF | REF | REF | REF |
|  | No, but careful | -1.5 (-4.4, 1.5) | 1.2 (-2.2, 4.7) | 3.6 (-0.9, 8.1) | 0.9 (-4.8, 6.7) | 1.5 (-3.7, 6.7) |
|  | Yes | -2.3 (-6.5, 2.0) | -3.4 (-7.2, 0.4) | -0.3 (-4.9, 4.2) | -0.5 (-5.6, 4.6) | -1.2 (-6.3, 3.8) |
| **Model 3** | No | REF | REF | REF | REF | REF |
|  | No, but careful | -0.9 (-3.8, 2.1) | 1.9 (-1.4, 5.2) | 3.9 (-0.5, 8.4) | 1.0 (-4.6, 6.6) | 1.6 (-3.4, 6.7) |
|  | Yes | -1.7 (-5.9, 2.4) | -3.4 (-7.1, 0.4) | 0.6 (-3.9, 5.1) | -0.2 (-5.2, 4.7) | 0.1 (-5.0, 5.1) |
| **Model 4** | No | REF | REF | REF | REF | REF |
|  | No, but careful | -0.6 (-3.5, 2.3) | 1.9 (-1.4, 5.2) | 3.7 (-0.7, 8.1) | 1.2 (-4.4, 6.8) | 0.7 (-4.3, 5.7) |
|  | Yes | -0.7 (-4.8, 3.5) | -3.6 (-7.4, 0.3) | 0.2 (-4.3, 4.7) | 0.1 (-4.9, 5.1) | -1.4 (-6.4, 3.6) |
|  | P_trend_ | 0.91 | 0.02* | 0.14 | 0.86 | 0.61 |
| β =beta coefficient, CI=confidence interval.  Model 1 adjusted for age. Model 2: model 1 + adjusted for marital status, number of people in the household. Model 3: model 2 + adjusted for smoking status, physical activity, energy intake. Model 4: adjusted for model 3 + presence of one or more chronic disease, body mass index.  * *P*≤0.05, ***P*≤0.01, ****P*≤0.001 | | | | | | |

**Supplementary Table S7:** Beta-coefficients (95% confidence intervals) from adjusted linear regression models for association between DHD15-Index and financial difficulty status in women

|  | | **Dutch**  β (95% CI) | **South-Asian Surinamese**  β (95% CI) | **African Surinamese**  β (95% CI) | **Turkish**  β (95% CI) | **Moroccan**  β (95% CI) |
| --- | --- | --- | --- | --- | --- | --- |
| **Model 1** | No | REF | REF | REF | REF | REF |
|  | No, but careful | -0.1 (-2.5, 2.3) | 2.7 (-0.6, 6.1) | -0.1 (-3.2, 3.1) | -2.3 (-7.1, 2.5) | 5.0 (1.2, 8.7)** |
|  | Yes | -3.0 (-6.2, 0.1) | -1.6 (-4.9, 1.7) | -0.6 (-3.6, 2.5) | -4.6 (-8.9, -0.2)* | 0.1 (-3.6, 3.9) |
| **Model 2** | No | REF | REF | REF | REF | REF |
|  | No, but careful | -0.0 (-2.4, 2.3) | 3.1 (-0.2, 6.4) | -0.0 (-3.2, 3.1) | -2.0 (-6.8, 2.7) | 4.8 (1.0, 8.6)* |
|  | Yes | -3.0 (-6.2, 0.2) | -0.7 (-4.0, 2.7) | -0.5 (-3.6, 2.6) | -4.0 (-8.4, 0.4) | 0.2 (-3.6, 3.9) |
| **Model 3** | No | REF | REF | REF | REF | REF |
|  | No, but careful | 0.5 (-1.8, 2.8) | 2.5 (-0.7, 5.8) | -0.1 (-3.2, 3.0) | -1.2 (-5.8, 3.5) | 4.7 (0.9, 8.4)* |
|  | Yes | -2.1 (-5.2, 1.0) | -0.5 (-3.8, 2.9) | -0.7 (-3.7, 2.4) | -3.0 (-7.3, 1.3) | 0.3 (-3.4, 4.1) |
| **Model 4** | No | REF | REF | REF | REF | REF |
|  | No, but careful | 0.6 (-1.7, 2.9) | 2.2 (-1.0, 5.5) | -0.2 (-3.3, 2.9) | -1.0 (-5.7, 3.7) | 4.8 (1.1, 8.6)* |
|  | Yes | -1.5 (-4.6, 1.7) | -1.0 (-4.4, 2.4) | -1.3 (-4.4, 1.9) | -2.2 (-6.6, 2.2) | 0.3 (-3.6, 4.1) |
|  | P_trend_ | 0.41 | 0.08 | 0.64 | 0.58 | 0.01* |
| β =beta coefficient, CI=confidence interval.  Model 1 adjusted for age. Model 2: model 1 + marital status, number of people in the household. Model 3: model 2 + adjusted for smoking status, physical activity, energy intake. Model 4: adjusted for model 3 + presence of one or more chronic disease, body mass index.  * *P*≤0.05, ***P*≤0.01, ****P*≤0.001 | | | | | | |

**Supplementary Table S8:** Sensitivity analysis for association between educational level and DHD15-Index for men

|  | | **Dutch**  β (95% CI) | **South-Asian Surinamese**  β (95% CI) | **African Surinamese**  β (95% CI) | **Turkish**  β (95% CI) | **Moroccan**  β (95% CI) |
| --- | --- | --- | --- | --- | --- | --- |
| **Model 5** | Higher | REF | REF | REF | REF | REF |
|  | Intermediate | -5.3 (-8.7, -1.8)** | 3.2 (-0.9, 7.4) | 0.6 (-4.4, 5.5) | -0.1 (-5.8, 5.6) | -0.2 (-5.7, 5.3) |
|  | Lower | -6.4 (-10.9, -2.0)** | -1.4 (-6.3, 3.5) | -0.8 (-6.2, 4.6) | -4.2 (-10.1, 1.8) | 1.6 (-4.8, 8.0) |
|  | Elementary | -5.1 (-14.3, 4.2) | -4.8 (-11.1, 1.5) | -6.9 (-16.4, 2.6) | -4.0 (-10.8. 2.9) | 4.8 (-2.0, 11.7) |
| **Model 6** | Higher | REF | REF | REF | REF | REF |
|  | Intermediate | -5.4 (-8.9, -1.9)** | 3.3 (-0.9, 7.5) | 0.6 (-4.4, 5.5) | -0.3 (-6.1, 5.4) | -0.1 (-5.7, 5.4) |
|  | Lower | -6.6 (-11.0, -2.1)** | -1.3 (-6.2, 3.6) | -0.8 (-6.2, 4.6) | -4.4 (-10.4, 1.6) | 1.7 (-4.7, 8.1) |
|  | Elementary | -5.2 (-14.5, 4.1) | -4.7 (-11.0, 1.5) | -7.0 (-16.4, 2.6) | -4.4 (-11.4, 2.6) | 5.0 (-1.9, 11.8) |
| β =beta coefficient, CI=confidence interval.  Model 5: adjusted for age, marital status, number of people in the household, smoking status, physical activity, energy intake, presence of one or more chronic disease, body mass index, occupational status.  Model 6: adjusted for age, marital status, number of people in the household, smoking status, physical activity, energy intake, presence of one or more chronic disease, body mass index, occupational status, financial difficulty status.  * *P*≤0.05, ***P*≤0.01, ****P*≤0.001 | | | | | | |

**Supplementary Table S9:** Sensitivity analysis for association between educational level and DHD15-Index for women

|  | | **Dutch**  β (95% CI) | **South-Asian Surinamese**  β (95% CI) | **African Surinamese**  β (95% CI) | **Turkish**  β (95% CI) | **Moroccan**  β (95% CI) |
| --- | --- | --- | --- | --- | --- | --- |
| **Model 5** | Higher | REF | REF | REF | REF | REF |
|  | Intermediate | -1.8 (-4.7, 1.1) | -0.9 (-4.7, 2.8) | -3.4 (-6.5, -0.4)* | -1.8 (-6.9, 3.2) | -3.8 (-8.1, 0.5) |
|  | Lower | -2.4 (-6.3, 1.4) | -0.8 (-5.0, 3.5) | -5.3 (-8.9, -1.6)** | -2.5 (-8.5, 3.5) | -2.8 (-8.1, 2.6) |
|  | Elementary | -7.8 (-15.8, 0.1) | -0.9 (-6.2, 4.5) | -5.1 (-12.1, 1.9) | -1.9 (-8.3, 4.5) | -2.4 (-8.1, 3.3) |
| **Model 6** | Higher | REF | REF | REF | REF | REF |
|  | Intermediate | -1.9 (-4.8, 1.1) | -0.9 (-4.7, 3.0) | -3.4 (-6.4, -0.4)* | -1.6 (-6.7, 3.5) | -3.8 (-8.2, 0.6) |
|  | Lower | -2.6 (-6.4, 1.3) | -0.6 (-4.9, 3.6) | -5.2 (-8.9, -1.6)** | -2.4 (-8.4, 3.6) | -2.8 (-8.3, 2.6) |
|  | Elementary | -8.1 (-16.1, -0.0)* | -0.7 (-6.1, 4.7) | -5.0 (-12.0, 2.0) | -1.6 (-8.1, 4.8) | -2.5 (-8.2, 3.3) |
| β =beta coefficient, CI=confidence interval.  Model 5: adjusted for age, marital status, number of people in the household, smoking status, physical activity, energy intake, presence of one or more chronic disease, body mass index, occupational status.  Model 6: adjusted for age, marital status, number of people in the household, smoking status, physical activity, energy intake, presence of one or more chronic disease, body mass index, occupational status, financial difficulty status.  * *P*≤0.05, ***P*≤0.01, ****P*≤0.001 | | | | | | |

**Supplementary Table S10:** Sensitivity analysis for association between occupational status and DHD15-Index for men

|  | | **Dutch**  β (95% CI) | **South-Asian Surinamese**  β (95% CI) | **African Surinamese**  β (95% CI) | **Turkish**  β (95% CI) | **Moroccan**  β (95% CI) |
| --- | --- | --- | --- | --- | --- | --- |
| **Model 5** | Higher | REF | REF | REF | REF | REF |
|  | Intermediate | -5.9 (-9.6, -2.2)** | 0.3 (-4.7, 5.3) | 1.2 (-4.7, 7.1) | -3.3 (-10.2, 3.6) | 0.0 (-7.0, 7.0) |
|  | Elementary | -4.5 (-9.3, 0.4) | 0.3 (-5.0, 5.6) | -0.8 (-7.1, 5.5) | -1.9 (-8.7, 4.8) | -4.8 (-11.9, 2.3) |
|  | Unknown/not in workforce | -4.7 (-11.4, 2.0) | -1.7 (-8.7, 5.4) | 0.6 (-7.3, 8.6) | -4.6 (-13.6, 4.3) | -9.9 (-20.0, 0.2) |
| **Model 6** | Higher | REF | REF | REF | REF | REF |
|  | Intermediate | -5.9 (-9.6, -2.2)** | 0.3 (-4.7, 5.3) | 1.2 (-4.7, 7.1) | -3.3 (-10.3, 3.6) | 0.2 (-6.9, 7.3) |
|  | Elementary | -4.6 (-9.5, 0.3) | 0.4 (-4.9, 5.8) | -0.9 (-7.2, 5.4) | -2.2 (-9.0, 4.7) | -4.5 (-11.7, 2.6) |
|  | Unknown/not in workforce | -4.7 (-11.4, 2.1) | -1.2 (-8.3, 5.9) | 0.6 (-7.4, 8.5) | -4.8 (-13.8, 4.2) | -9.8 (-19.9, 0.4) |
| β =beta coefficient, CI=confidence interval.  Model 5: adjusted for age, marital status, number of people in the household, smoking status, physical activity, energy intake, presence of one or more chronic disease, body mass index, educational level.  Model 6: adjusted for age, marital status, number of people in the household, smoking status, physical activity, energy intake, presence of one or more chronic disease, body mass index, educational level, financial difficulty status.  * *P*≤0.05, ***P*≤0.01, ****P*≤0.001 | | | | | | |

**Supplementary Table S11:** Sensitivity analysis for association between occupational status and DHD15-Index for women

|  | | **Dutch**  β (95% CI) | **South-Asian Surinamese**  β (95% CI) | **African Surinamese**  β (95% CI) | **Turkish**  β (95% CI) | **Moroccan**  β (95% CI) |
| --- | --- | --- | --- | --- | --- | --- |
| **Model 5** | Higher | REF | REF | REF | REF | REF |
|  | Intermediate | -0.5 (-3.7, 2.7) | -2.4 (-7.0, 2.2) | 2.2 (-1.7, 6.2) | -2.8 (-9.5, 3.8) | -4.9 (-10.1, 0.3) |
|  | Elementary | -3.8 (-7.8, 0.1) | -5.3 (-10.2, -0.4)* | 0.7 (-3.9, 5.2) | -4.0 (-10.7, 2.7) | -8.6 (-14.1, -3.1)** |
|  | Unknown/not in workforce | -6.9 (-11.9, -1.9)** | -5.3 (-11.3, 0.7) | -0.2 (-5.9, 5.4) | -1.8 (-8.9, 5.4) | -7.2 (-12.8, -1.6)* |
| **Model 6** | Higher | REF | REF | REF | REF | REF |
|  | Intermediate | -0.6 (-3.8, 2.6) | -2.4 (-7.0, 2.2) | 2.3 (-1.7, 6.2) | -2.8 (-9.4, 3.9) | -5.0 (-10.2, 0.2) |
|  | Elementary | -3.9 (-7.8, 0.1) | -5.3 (-10.2, -0.3)* | 0.7 (-3.8, 5.3) | -3.8 (-10.5, 3.0) | -8.6 (-14.1, -3.1)** |
|  | Unknown/not in workforce | -6.9 (-11.9, -1.9)** | -5.3 (-11.3, 0.7) | -0.2 (-5.9, 5.4) | -1.6 (-8.7, 5.5) | -7.2 (-12.8, -1.6)* |
| β =beta coefficient, CI=confidence interval.  Model 5: adjusted for age, marital status, number of people in the household, smoking status, physical activity, energy intake, presence of one or more chronic disease, body mass index, educational level.  Model 6: adjusted for age, marital status, number of people in the household, smoking status, physical activity, energy intake, presence of one or more chronic disease, body mass index, educational level, financial difficulty status.  * *P*≤0.05, ***P*≤0.01, ****P*≤0.001 | | | | | | |

**Supplementary Table S12:** Sensitivity analysis for association between financial difficulty status and DHD15-Index for men

|  | | **Dutch**  β (95% CI) | **South-Asian Surinamese**  β (95% CI) | **African Surinamese**  β (95% CI) | **Turkish**  β (95% CI) | **Moroccan**  β (95% CI) |
| --- | --- | --- | --- | --- | --- | --- |
| **Model 5** | No | REF | REF | REF | REF | REF |
|  | No, but careful | 0.5 (-2.4. 3.3) | 1.9 (-1.3, 5.2) | 3.6 (-0.8, 8.1) | 1.9 (-3.8. 7.7) | 1.1 (-3.9, 6.1) |
|  | Yes | 0.9 (-3.2, 5.0) | -3.2 (-7.0, 0.6) | 0.7 (-3.9, 5.3) | 1.6 (-3.6, 6.9) | -1.2 (-6.2, 3.9) |
| **Model 6** | No | REF | REF | REF | REF | REF |
|  | No, but careful | 0.7 (-2.1, 3.6) | 1.9 (-1.3, 5.2) | 3.7 (-0.8, 8.2) | 2.0 (-3.8, 7.7) | 1.0 (-4.0, 6.0) |
|  | Yes | 1.0 (-3.0, 5.1) | -3.2 (-7.0, 0.6) | 0.7 (-3.9, 5.4) | 1.9 (-3.4, 7.2) | -0.9 (-5.8, 4.1) |
| β =beta coefficient, CI=confidence interval.  Model 5: adjusted for age, marital status, number of people in the household, smoking status, physical activity, energy intake, presence of one or more chronic disease, body mass index, educational level.  Model 6: adjusted for age, marital status, number of people in the household, smoking status, physical activity, energy intake, presence of one or more chronic disease, body mass index, educational level, occupational status.  * *P*≤0.05, ***P*≤0.01, ****P*≤0.001 | | | | | | |

**Supplementary Table S13:** Sensitivity analysis for association between financial difficulty status and DHD15-Index for women

|  | | **Dutch**  β (95% CI) | **South-Asian Surinamese**  β (95% CI) | **African Surinamese**  β (95% CI) | **Turkish**  β (95% CI) | **Moroccan**  β (95% CI) |
| --- | --- | --- | --- | --- | --- | --- |
| **Model 5** | No | REF | REF | REF | REF | REF |
|  | No, but careful | 1.6 (-0.7, 3.9) | 2.3 (-0.9, 5.6) | 0.0 (-3.1, 3.1) | -1.1 (-5.9, 3.6) | 5.0 (1.3, 8.8)** |
|  | Yes | -0.2 (-3.3, 3.0) | -0.5 (-3.9, 3.0) | -0.3 (-3.5, 2.9) | -2.1 (-6.5, 2.4) | 1.3 (-2.6, 5.2) |
| **Model 6** | No | REF | REF | REF | REF | REF |
|  | No, but careful | 1.7 (-0.6, 4.0) | 2.4 (-0.9, 5.6) | -0.0 (-3.1, 3.1) | -1.1 (-5.9, 3.6) | 4.9 (1.1, 8.6)* |
|  | Yes | 0.1 (-3.1, 3.2) | -0.4 (-3.9, 3.0) | -0.3 (-3.5, 2.9) | -2.0 (-6.5, 2.5) | 1.2 (-2.7, 5.0) |
| β =beta coefficient, CI=confidence interval.  Model 5: adjusted for age, marital status, number of people in the household, smoking status, physical activity, energy intake, presence of one or more chronic disease, body mass index, educational level.  Model 6: adjusted for age, marital status, number of people in the household, smoking status, physical activity, energy intake, presence of one or more chronic disease, body mass index, educational level, occupational status.  * *P*≤0.05, ***P*≤0.01, ****P*≤0.001 | | | | | | |

**Supplementary Table S14:** Age-adjusted median (lower quartile, upper quartile) DHD15-Index by ethnicity in men

| **Dietary Component** | **Dutch**  (n=633) | **South-Asian Surinamese**  (n=395) | **African Surinamese**  (n=298) | **Turkish**  (n=273) | **Moroccan**  (n=258) | **Pearson’s F Statistic**  **(*P*-value)** |
| --- | --- | --- | --- | --- | --- | --- |
| Vegetables | 7.7  (5.2, 9.8) | 5.7  (3.2, 8.9) | 6.6  (4.0, 9.7) | 6.5  (3.8, 9.8) | 5.5  (2.6, 9.3) | 9.51  (<0.0001) |
| Fruit | 5.1  (1.9, 10.0) | 5.9  (2.6, 10.0) | 6.0  (2.6, 10.0) | 8.1  (3.4, 10.0) | 8.0  (4.0, 10.0) | 12.46  (<0.0001) |
| Wholegrains | 5.6  (5.1, 6.8) | 4.9  (2.6, 5.0) | 4.6  (1.6, 5.0) | 5.2  (4.2, 6.0) | 5.4  (5.0, 6.3) | 20.65  (<0.0001) |
| Legumes | 10.0  (2.9, 10.0) | 10.0  (3.6, 10.0) | 9.4  (3.4, 10.0) | 10.0  (9.9, 10.0) | 10.0  (9.5, 10.0) | 4.45  (0.001) |
| Nuts & seeds | 8.8  (3.5, 10.0) | 8.6  (2.3, 10.0) | 9.2  (2.5, 10.0) | 7.3  (3.1, 10.0) | 7.3  (2.5. 10.0) | 1.83  (0.12) |
| Dairy | 6.5  (3.1, 9.0) | 4.5  (1.6, 8.1) | 4.4  (1.9, 7.4) | 5.3  (2.3, 8.6) | 6.1  (2.6, 9.4) | 9.18  (<0.0001) |
| Fish | 5.3  (2.7, 9.6) | 7.9  (2.7, 10.0) | 4.9  (2.7, 10.0) | 2.9  (1.4, 8.0) | 4.3  (2.7, 10.0) | 10.19  (<0.0001) |
| Tea | 3.5  (0.6, 9.8) | 4.1  (0.8, 7.6) | 3.2  (1.0, 7.5) | 8.0  (3.8, 10.0) | 6.0  (1.8, 9.8) | 16.56  (<0.0001) |
| Cooking fats & oils | 10.0  (1.3, 10.0) | 10.0  (9.9, 10.0) | 10.0  (2.3, 10.0) | 3.9  (1.4, 10.0) | 10.0  (5.1, 10.0) | 74.36  (<0.0001) |
| Red meat | 8.8  (3.2, 10.0) | 10.0  (9.6, 10.0) | 10.0  (7.2, 10.0) | 6.0  (0.3, 10.0) | 8.8  (0.5, 10.0) | 18.64  (<0.0001) |
| Processed meat | 6.3  (3.2, 8.6) | 9.2  (7.4, 10.0) | 9.2  (7.1, 10.0) | 9.4  (8.0, 10.0) | 9.9  (9.0, 10.0) | 122.69  (<0.0001) |
| SSBs & fruit juices | 4.7  (0.0, 8.4) | 3.3  (0.0, 7.4) | 1.0  (0.0, 5.9) | 7.6  (1.3, 9.2) | 5.3  (0.0, 8.8) | 26.04  (<0.0001) |
| Alcohol | 7.7  (1.3, 10.0) | 10.0  (10.0, 10.0) | 10.0  (9.9, 10.0) | 10.0  (10.0, 10.0) | 10.0  (10.0, 10.0) | 34.72  (<0.0001) |
| SSBs=sugar sweetened beverages | | | | | | |

**Supplementary Table S15:** Age-adjusted median (lower quartile, upper quartile) DHD15-Index by ethnicity in women

| **Dietary Component** | **Dutch**  (n=789) | **South-Asian Surinamese**  (n=576) | **African Surinamese**  (n=646) | **Turkish**  (n=305) | **Moroccan**  (n=429) | **Pearson’s F Statistic**  **(*P*-value)** |
| --- | --- | --- | --- | --- | --- | --- |
| Vegetables | 8.6  (5.8, 10.0) | 7.0  (4.4, 10.0) | 7.7  (4.8, 8.2) | 8.8  (5.8, 10.0) | 7.4  (4.2, 10.0) | 10.39  (<0.0001) |
| Fruit | 7.5  (3.5, 10.0) | 7.9  (3.8, 10.0) | 8.0  (3.3, 10.0) | 8.5  (4.8, 10.0) | 8.3  (4.4, 10.0) | 1.11  (0.35) |
| Wholegrains | 5.6  (5.0, 6.9) | 4.4  (2.6, 5.1) | 3.8  (1.7, 5.0) | 5.3  (3.7, 6.0) | 5.3  (4.3, 5.9) | 79.86  (<0.0001) |
| Legumes | 10.0  (2.8, 10.0) | 10.0  (3.5, 10.0) | 9.4  (2.5, 10.0) | 10.0  (10.0, 10.0) | 10.0  (8.0, 10.0) | 1.31  (0.27) |
| Nuts & seeds | 7.1  (2.8, 10.0) | 6.3  (2.3, 10.0) | 4.6  (1.4, 10.0) | 5.3  (2.0, 10.0) | 4.9  (1.7, 1.0) | 7.86  (<0.0001) |
| Dairy | 6.2  (3.2, 9.1) | 4.5  (1.8, 8.3) | 4.1  (1.4, 7.6) | 4.7  (2.3, 8.1) | 5.5  (2.4, 8.7) | 13.50  (<0.0001) |
| Fish | 5.0  (2.7, 8.4) | 7.7  (2.7, 10.0) | 5.3  (2.7, 10.0) | 3.1  (0.0, 7.1) | 3.5  (2.7, 8.4) | 22.32  (<0.0001) |
| Tea | 10.0  (3.8, 10.0) | 7.6  (2.2, 10.0) | 7.6  (2.7, 10.0) | 8.1  (5.4, 10.0) | 6.5  (2.2, 10.0) | 21.46  (<0.0001) |
| Cooking fats & oils | 10.0  (1.2, 10.0) | 10.0  (9.9, 10.0) | 10.0  (3.3, 10.0) | 4.1  (1.2, 10.0) | 10.0  (9.0, 10.0) | 55.44  (<0.0001) |
| Red meat | 10.0  (8.6, 10.0) | 10.0  (10.0, 10.0) | 10.0  (10.0, 10.0) | 10.0  (2.3, 10.0) | 10.0  (6.4, 10.0) | 0.00  (1.00) |
| Processed meat | 8.3  (6.3, 9.5) | 9.8  (8.7, 10.0) | 9.5  (7.9, 10.0) | 9.7  (9.2, 10.0) | 10.0  (9.6, 10.0) | 118.39  (<0.0001) |
| SSBs & fruit juice | 6.9  (2.5, 9.0) | 6.4  (1.8, 8.9) | 4.3  (0.2, 8.4) | 8.7  (6.1, 9.8) | 7.9  (3.2, 9.7) | 24.35  (<0.0001) |
| Alcohol | 10.0  (1.4, 10.0) | 10.0  (10.0, 10.0) | 10.0  (10.0, 10.0) | 10.0  (10.0, 10.0) | 10.0  (10.0, 10.0) | 0.00  (1.00) |
| SSBs=sugar sweetened beverages | | | | | | |
